# SegCycle-SPADE: An end-to-end framework for semantic segmentation-based automated extraction and artistic reconstruction of traditional craft patterns using conditional GAN

**DOI:** 10.1371/journal.pone.0329100

**Published:** 2025-11-18

**Authors:** Bei Huang, Lequn Mo

**Affiliations:** 1 School of Art and Design, Guangdong University of Technology, Guangzhou, China; 2 Information College of Guangdong Communication Polytechnic, Guangzhou, China; Chongqing Normal University, CHINA

## Abstract

This paper addresses the challenge of traditional handicraft pattern extraction and generation, focusing on accurate segmentation and high-quality pattern reconstruction. We propose the SegCycle-SPADE model, combining SegFormer for semantic segmentation, CycleGAN for pattern generation, and SPADE for style transfer, to achieve a balance between segmentation accuracy, generation quality, and inference efficiency. Experiments on datasets such as Batik Nitik 960, Fashion-MNIST, and DeepFashion show that SegCycle-SPADE outperforms baseline models like U-Net and DeepLabV3+ with significant improvements in PA (88.6%), mIoU (78.1%), and Boundary F1 (73.8%). In terms of pattern generation, SegCycle-SPADE also achieves superior results in PSNR (27.8 dB), SSIM (0.89), and FID (34.2), outperforming Pix2Pix, Stable Diffusion, and other models. The model demonstrates its potential for the digital regeneration of traditional handicraft patterns, offering a robust solution for high-quality and efficient pattern generation, with substantial contributions to digital cultural heritage preservation and innovation.

## Introduction

Traditional craft patterns, as dynamic carriers of human civilization, contain unique cultural memories and artistic value. From the intricate totems of Miao silverware to the geometric patterns of Indonesian batik, each pattern carries the history and aesthetics of a specific region [1]. However, with the acceleration of modern industrialization, traditional crafts face the crisis of lost inheritance and pattern extinction [2]. Digital preservation technologies, through the automatic extraction and reconstruction of patterns, provide a new path for the sustainable inheritance of cultural heritage [3]. However, the high complexity and diversity of traditional craft patterns, such as the three-dimensional texture of embroidery and the glaze color gradients on ceramics, pose significant challenges to automated processing technologies.

In recent years, deep learning has made significant progress in the field of traditional handicraft pattern processing. In the area of semantic segmentation, convolutional neural network (CNN)-based models such as U-Net and DeepLab series have laid the foundation for pattern extraction by achieving pixel-level classification through encoder-decoder structures and dilated convolutions [4–6]. Emerging Transformer architectures (such as SETR and Segmenter) enhance global context modeling capabilities with self-attention mechanisms, showing excellent performance in complex texture segmentation [7,8]. In the area of image generation and style transfer, classic models like CycleGAN enable unpaired image style transfer, StarGAN supports multi-domain generation, and diffusion-based models like Stable Diffusion have become research hotspots due to their high-fidelity image generation abilities [9,10]. However, there are still key limitations when applying existing technologies to traditional handicraft patterns: In segmentation, CNNs are limited by local receptive fields, making it difficult to capture subtle differences in embroidery stitches and complex contours in bronzeware patterns [11,12]. Although Transformer models improve global perception, their computational cost is high, and their performance on small-object segmentation is suboptimal. In the generation process, GANs are prone to mode collapse and gradient vanishing, leading to missing pattern details or distorted styles [13–15]. Diffusion models can generate high-quality images but struggle to precisely capture the structure and artistic features of traditional patterns, and they lack generalization ability across cultures and craft styles [16,17]. Additionally, while 3D vision technologies have made progress in complex scenarios (such as cultural heritage damage recognition), integrated research on “semantic precise segmentation - stylized generation” for 2D traditional patterns remains a breakthrough yet to be achieved [18,19].

To address these issues, this paper proposes the SegCycle-SPADE model—an end-to-end framework based on semantic segmentation and conditional GAN, designed for automatic extraction and artistic reconstruction of traditional handicraft patterns [20,21]. Compared to existing research, the core innovation of this study lies in breaking through the limitations of “segmentation and generation module decoupling” by dynamically linking SegFormer [22] (a layered Transformer semantic segmentation module) with CycleGAN (a generative adversarial module) through SPADE (Spatially Adaptive Normalization), allowing the semantic segmentation results to directly guide the style and structure constraints of the generation process. Specifically, the model operates through three core modules: 1) The SegFormer module, using a layered Transformer encoder and a lightweight MLP decoder, accurately captures the global semantic structure and local texture details of the pattern, achieving multi-scale feature extraction; 2) The CycleGAN module [10], using the adversarial game between the generator and discriminator, combined with the unpaired image translation characteristic, completes the style transfer and high-quality generation of traditional patterns; 3) The SPADE module [23], which takes the segmentation mask output from SegFormer as a conditional input and dynamically adjusts the normalization parameters of the CycleGAN generator, deeply binding the generation process with the semantic regions of the pattern, ensuring a balance between structural accuracy and artistic style authenticity in the reconstructed pattern.

The main contributions of this paper are as follows:

- Propose a cross-module deep coupling mechanism, dynamically associating SegFormer’s semantic segmentation results with CycleGAN’s generation process via Spatially-Adaptive Denormalization (SPADE), constructing a “semantic region - generation strategy” adaptive matching framework, addressing the structural distortion issues caused by the decoupling of segmentation and generation modules in traditional methods.
- Achieve multi-scale feature collaborative optimization by leveraging SegFormer’s global semantic capture ability with the fine-grained generation of local textures through CycleGAN’s adversarial game, forming a “global structure - local detail” complementary pattern, enhancing the integrity and artistic detail performance of traditional pattern reconstruction.
- Build an unpaired data-driven cross-domain processing paradigm, utilizing CycleGAN’s unpaired image transformation properties and SPADE’s unsupervised conditional constraints. The model can handle traditional patterns from different cultural backgrounds and craft styles without paired annotated data, providing a lightweight solution for cross-cultural pattern transfer.

## Related works

In the field of automated extraction and artistic reconstruction of traditional craft patterns, numerous deep learning and artificial intelligence methods have been proposed, covering tasks such as semantic segmentation, image generation, and style transfer. These methods are typically based on architectures like Convolutional Neural Networks (CNNs), Generative Adversarial Networks (GANs), and Transformers, and have made significant progress. However, most existing models still face challenges such as detail loss, high computational cost, and module decoupling, especially in terms of balancing the artistic quality and structural accuracy of generated images. Table 1 summarizes the key models proposed in recent years in this field and compares them across various aspects.

**Table 1:** Comparison of major models proposed in recent years for traditional craft pattern extraction and reconstruction, including model type, application tasks, advantages, and shortcomings.

Model	Application Task	Advantages	Shortcomings
U-Net [24]	Pattern Segmentation	High pixel-level segmentation accuracy	Limited by local receptive fields, hard to capture complex details
DeepLabV3+ [25]	Semantic Segmentation	Enhanced dilated convolutions, suitable for complex texture segmentation	Poor performance on small object segmentation, high computational cost
SegFormer [7,26]	Semantic Segmentation	Strong global semantic modeling ability, excellent for complex patterns	High computational cost, difficult for real-time processing
CycleGAN [9,10,27]	Image Style Transfer, Pattern Generation	No paired data needed, capable of generating high-quality images	Mode collapse, loss of generated details
StarGAN [28,29]	Multi-domain Generation	Supports generation of multiple styles	Difficulty balancing quality and diversity of generated images
Pix2Pix [30–32]	Image Translation	Generates high-quality images, suitable for paired data	Poor retention of pattern details
SPADE [23,33]	Image Generation	Spatially-adaptive normalization, generates high-fidelity images	High computational cost, slow generation process
SETR [34,35]	Image Segmentation	Strong global context modeling ability	Lacks precise modeling at the detail level
Segmenter [36]	Semantic Segmentation	Self-attention mechanism enhances global perception	High computational demand, large memory usage
Stable Diffusion [37,38]	Image Generation	High-quality image generation capability	Difficult to control pattern structure and artistic features
VAE-GAN [39,40]	Image Generation and Reconstruction	Combines variational autoencoders’ latent space learning, enhances diversity	Generation quality may not be as good as pure GAN methods
FPN [41]	Object Detection and Segmentation	Enhanced multi-scale feature extraction, adapts to different patterns	Does not preserve details as well as GAN models
GAN-INT [42,43]	Image Generation	Improves detail quality in generated images	Mode collapse, unstable

From the table, it is evident that while existing techniques have made significant progress in pattern generation and segmentation for traditional crafts, many challenges and shortcomings remain. CNNs and GANs perform well in detail retention and generation quality but still face issues like local receptive field limitations, mode collapse, and instability in generation [44]. Transformer architectures, such as SegFormer and Segmenter, excel at capturing global semantics and context but come with high computational costs and struggle with fine-grained detail. Diffusion models like Stable Diffusion offer high-quality generation capabilities but lack precise control over the structure and artistic features of the patterns. Moreover, most existing methods use independent training approaches, lacking effective information exchange and collaborative optimization between segmentation and generation modules, which leads to a failure to balance pattern structural accuracy with artistic style authenticity [37].

To address these limitations, the SegCycle-SPADE model proposed in this paper introduces the Spatially-Adaptive Denormalization (SPADE) module, which enables deep coupling between the semantic segmentation and generation modules, addressing the issues of segmentation and generation decoupling, as well as detail loss in traditional methods. This results in improved performance in the automated extraction and artistic reconstruction of traditional craft patterns.

## Methodology

### SegCycle-SPADE model overview

**Fig 1:**
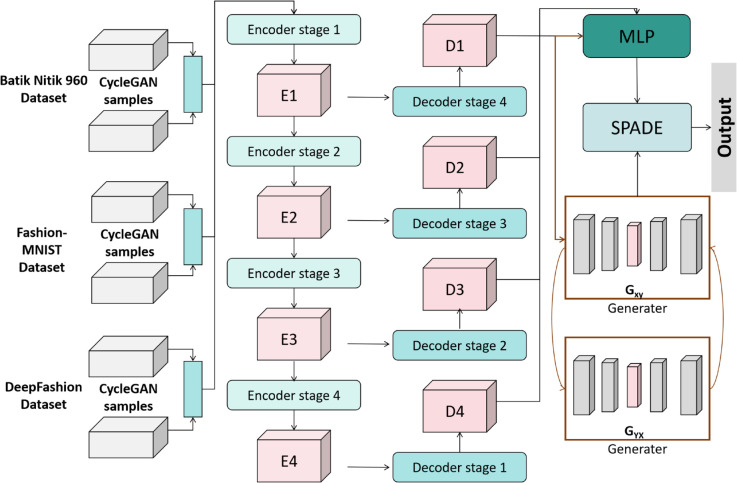
End-to-end architecture and workflow of the SegCycle-SPADE model.

The complexity of traditional handicraft patterns, such as the intricate hollow patterns of Miao silver jewelry and the gradient colors of Dunhuang murals, poses significant challenges to automated extraction and reconstruction technologies. Existing methods struggle to simultaneously ensure the precision of pattern structures and the authenticity of artistic styles. To address this, we propose the SegCycle-SPADE model, constructing an “Semantic Segmentation - Conditional Generation - Joint Optimization” end-to-end closed-loop framework (Fig 1), enabling efficient processing of traditional handicraft patterns.

As shown in Fig 1, the SegCycle-SPADE model consists of three core modules. The input traditional handicraft pattern image first enters the SegFormer semantic segmentation module. This module leverages a layered Transformer encoder and a lightweight MLP decoder to capture multi-scale semantic features from the image using self-attention mechanisms. It performs pixel-level classification of pattern elements, such as texture areas, contours, and backgrounds, producing a semantic segmentation mask that provides the structural foundation for subsequent processing.

After the segmentation mask is generated, it is concatenated with the original image features and used as conditional input for the CycleGAN generation module. CycleGAN utilizes an adversarial training mechanism between a generator and a discriminator. Guided by the segmentation mask, the generator reconstructs the pattern or performs style transfer, generating specific details based on the region information in the mask. The discriminator ensures that the generated patterns adhere to real-world distributions through adversarial training.

Finally, the SPADE joint optimization module performs spatially adaptive normalization on the CycleGAN generation process based on the segmentation mask. SPADE maps the mask information into normalization parameters via an MLP, dynamically adjusting the activation distribution of the generator’s convolution layers. During this process, the model simultaneously optimizes the adversarial loss, cycle consistency loss, and perceptual loss. The adversarial loss ensures the visual authenticity of the generated pattern; the cycle consistency loss enforces structural consistency between the generated image and the original; and the perceptual loss ensures that the generated pattern aligns with the artistic style and traditional craftsmanship features. Through the collaborative optimization of these three losses, the model ultimately outputs a high-precision reconstructed pattern, achieving a balance between structural accuracy and artistic fidelity.

### SegFormer module

**Fig 2:**
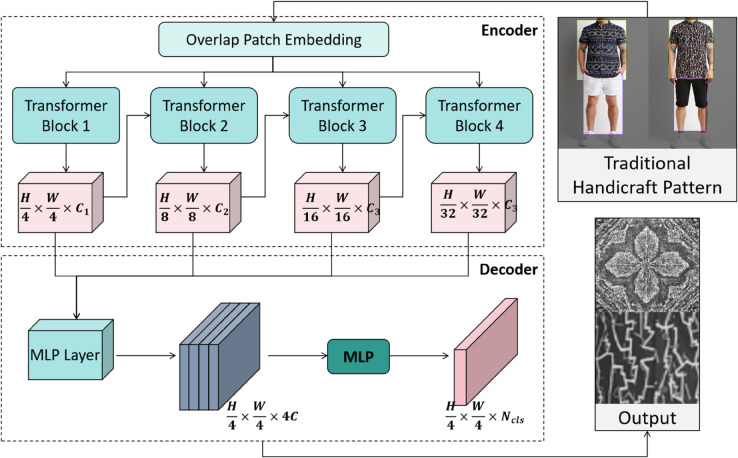
Semantic segmentation module architecture: A layered Transformer encoder and lightweight MLP decoder design based on SegFormer.

In the SegCycle-SPADE model, the SegFormer semantic segmentation module (Fig 2) plays the core role of extracting precise semantic structures from the input image. The segmentation mask output by this module serves as the conditional input to the subsequent CycleGAN generation module, directly determining the structural accuracy of the traditional handicraft pattern reconstruction. The design of this module deeply aligns with the model’s “semantic parsing - conditional generation - joint optimization” closed-loop logic. By performing pixel-level segmentation of semantic elements such as embroidery stitch distribution and ceramic pattern contours, it provides interpretable spatial constraints for the generation module, preventing the pattern distortion commonly seen in traditional generative models due to a lack of structural guidance. Its architectural innovation stems from the dual challenges posed by traditional handicraft patterns: capturing the global topological structure of silver jewelry’s hollow patterns while also locating the fine textures of wax dyeing ice cracks [7,22]. To address this, a collaborative design of the layered Transformer encoder and lightweight MLP decoder is adopted, optimizing computational efficiency while ensuring global semantic modeling capability.

The encoder adopts a four-level Swin Transformer architecture, using an alternating iteration of sliding window and shifted window self-attention mechanisms to extract multi-scale features from traditional patterns [45]. The key advantage of this hierarchical design is that it reduces the self-attention computational complexity from *O*(*H*^2^*W*^2^) to *O*(*HW*), enabling efficient processing of global context relationships in complex patterns such as Miao brocade. The feature update process for the *l*-th layer encoder is represented as:

$$F^{l}=\text{SW-MSA}(\text{LN}(F^{l-1}))+F^{l-1}$$ (1)

$$F^{l}=\text{FFN}(\text{LN}(F^{l}))+F^{l}$$ (2)

where *F*${\;}^{\text{l}-1}$ is the input feature map, corresponding to the original visual information in traditional patterns; SW-MSA(·) captures long-range dependencies (e.g., the continuous coiling dragon patterns in bronze vessel motifs) via the shifted window mechanism; LN(·) is layer normalization; and FFN(·) is a feed-forward neural network. For example, in the case of Dunhuang murals, this structure simultaneously models the global dynamic contours of flying celestial beings and the local details of the folds in their clothing.

The multi-head self-attention mechanism enhances semantic correlation modeling by computing relationships in parallel across multiple subspaces:

$$\text{Attention}(Q,K,V)=\text{Softmax}\left(\frac{QK^{T}}{\sqrt{d_{k}}}\right)V$$ (3)

where *Q*, *K*, and *V* correspond to the regions to be segmented (e.g., silver jewelry edges), contextually related areas (e.g., hollow background), and feature vectors, respectively; *d*_*k*_ is the key vector dimension. This mechanism avoids the limitations of CNN’s local receptive fields, ensuring that nested geometric patterns in paper-cut designs are not broken during segmentation.

To integrate multi-level semantic information from traditional patterns, the encoder employs a feature pyramid fusion strategy to complement coarse and fine-grained features:

$$F_{i}^{\prime }=\text{Concat}(F_{i},\text{Upsample}(F_{i+1}))$$ (4)

where *F*_*i*_ is the coarse-grained feature map output by the *i*-th encoder (e.g., the overall partitioning of embroidery patterns), and *F*_*i* + 1_ is the fine-grained feature map from the next level (e.g., stitch direction). The Upsample(·) operation performs upsampling using bilinear interpolation. For example, in ceramic blue-and-white patterns, this mechanism combines high-level features of the intertwined branch pattern’s overall layout with low-level features of the glaze transition at the petal edges, ensuring that the segmentation result retains both the topological structure and the color boundary.

The lightweight MLP decoder (Fig 2) maps the fused features into the segmentation mask using pointwise convolutions and bilinear interpolation:

$$F_{\text{fusion}}=\sum_{i=1}^{4}{\text{MLP}}_{i}(F_{i}^{\prime })$$ (5)

$$P=\text{Softmax}(\text{FC}(F_{\text{fusion}}))$$ (6)

where ${\text{MLP}}_{i}(\cdot )$ is the MLP dedicated to each feature level, and FC(·) is a fully connected layer. This design reduces the parameter count by 62

$$L_{\text{seg}}=L_{\text{CE}}+\lambda L_{\text{Dice}}$$ (7)

where the cross-entropy loss $L_{\text{CE}}$ measures the difference in class distribution:

$$L_{\text{CE}}=-\frac{1}{N}\sum_{i=1}^{N}\sum_{c=1}^{C}y_{i,c}\log P_{i,c}$$ (8)

The Dice loss $L_{\text{Dice}}$ specifically optimizes the segmentation of small targets (e.g., detailed engravings on silver jewelry):

$$L_{\text{Dice}}=1-\frac{2\sum_{i=1}^{N}P_{i}y_{i}+\epsilon }{\sum_{i=1}^{N}P_{i}^{2}+\sum_{i=1}^{N}y_{i}^{2}+\epsilon }$$ (9)

where *N* is the total number of pixels, *C* is the number of semantic categories (e.g., texture region, contour, background); *y*_*i*,*c*_ is the true label for pixel *i*; *P*_*i*,*c*_ is the predicted probability; ϵ=1e−5 is a smoothing factor, and λ=0.8 is a balancing coefficient.

The segmentation mask output by SegFormer not only contains pixel-level annotations for each semantic category but also preserves the structural topological relationships of traditional patterns (e.g., the connectivity of wax dyeing cracks and the directional flow of embroidery stitches). This information is fed into the CycleGAN generation module via feature concatenation, enabling the generator to process the patterns based on semantic regions—such as enhancing edge clarity in the “contour region” and generating details consistent with traditional craftsmanship in the “texture region,” thereby achieving collaborative control of pattern structure and style.

### CycleGAN module

**Fig 3:**
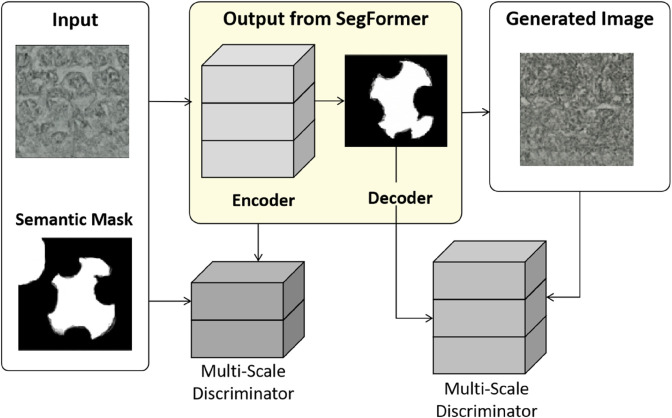
Semantic conditional generator and multi-scale discriminator design based on CycleGAN for traditional handicraft pattern generation.

In the SegCycle-SPADE model, the CycleGAN generation module (Fig 3) is responsible for transforming the semantic masks output by SegFormer into high-quality traditional handicraft patterns. This module is based on an unpaired image-to-image translation framework [10], with the core function being to perform style transfer and artistic reconstruction of traditional patterns under the constraint of the semantic segmentation mask. For example, it can convert the segmentation mask of Miao silver jewelry into a wax dyeing style pattern or complete the structure of damaged embroidery patterns. Unlike traditional CycleGAN, this module introduces semantic conditional inputs and multi-scale dilated convolutions to address the issues of structural distortion and detail loss commonly encountered in traditional generative models when handling complex patterns. Its architectural design is deeply aligned with the generation requirements of traditional handicraft patterns, which prioritize “structural semantics” over “artistic style.”

The generator adopts an encoder-decoder architecture, with a unique feature where the semantic mask *M* output by SegFormer is concatenated with the original image features *I* as input, forming a “semantic-visual” dual conditional constraint. The forward propagation process of the generator can be represented as:

G(I,M)=Decoder(Encoder([I;M])) (10)

where [*I;M*] denotes the feature concatenation operation. The encoder part extracts semantic-visual features through 7 residual blocks, with each residual block defined as:

R(x)=x+ConvReLU(Norm(Conv(x))) (11)

where Conv(·) represents the convolution operation, Norm(·) is Instance Normalization, and ConvReLU(·) represents convolution followed by the ReLU activation function. This design effectively captures structural information from the semantic mask (such as the contours of the hollow regions in silver jewelry) and the style features from the original image (such as the blue-and-white color scheme in wax dyeing).

To enhance the generator’s ability to produce fine details of traditional patterns, the decoder introduces multi-scale dilated convolutions [44], where the dilation rate *r* increases with each layer (e.g., r=1,2,4), expanding the receptive field from 3×3 to 15×15. The computation is represented as:

$$y[i]=\sum_{k=1}^{K}x[i+r\cdot k]\cdot w[k]$$ (12)

where *x* represents the input features, *w* is the convolution kernel weights, and *K* is the kernel size. This mechanism is particularly suited for generating traditional pattern elements with multi-scale textures, such as embroidery stitches or ceramic ice cracks.

The discriminator adopts the PatchGAN architecture, which forces the generator to output patterns rich in local details by determining the authenticity of 70×70 pixel patches in the generated image. The objective function of the discriminator is:

$$L_{GAN}(G,D)={𝔼}_{x\sim p_{\text{data}}(x)}[\log D(x)]+{𝔼}_{z\sim p_{z}(z)}[\log(1-D(G(z)))]$$ (13)

where *x* represents the real traditional pattern and *z* represents the input condition (semantic mask + image features). To enforce both global structural and local detail constraints, this module employs a multi-scale discriminator consisting of three discriminators $D_{1},D_{2},D_{3}$ corresponding to the original image, a half-scale image, and a quarter-scale image. The total adversarial loss is:

$$L_{multiGAN}=\sum_{i=1}^{3}L_{GAN}(G,D_{i})$$ (14)

This design allows the discriminator to supervise the authenticity of the overall framework of silver jewelry patterns while also constraining the quality of the generated local details, such as engraved textures.

To prevent the loss of semantic structure during generation, the module introduces the cycle consistency loss *L*_*cycle*_:

$$L_{cycle}={𝔼}_{x\sim p_{\text{data}}(x)}[\|G^{\prime }(G(x,M),M^{\prime })-x{\|}_{1}]$$ (15)

where $G^{\prime }$ is the reverse generator and $M^{\prime }$ is the reverse semantic mask. This loss ensures that the generated image remains consistent with the original pattern in terms of structure, such as preventing topological changes in crack directions during wax dyeing pattern generation.

Additionally, to preserve the artistic style of traditional handicraft patterns, the semantic perceptual loss *L*_*sem*_ is designed:

$$L_{sem}=\frac{1}{HWC}\sum_{h,w,c}\|\phi (G(x,M))-\phi (x){\|}_{2}^{2}$$ (16)

where ϕ(·) is the feature extraction function of a pre-trained VGG network, and the loss measures the difference in high-level semantic features (such as embroidery stitch patterns and ceramic decoration types) between the generated and real patterns, ensuring the authenticity of the artistic style in the generated result.

The CycleGAN generation module, through the “semantic conditional input - multi-scale generation - multi-loss supervision” architecture design, achieves both structural fidelity and style transfer for traditional handicraft patterns. This module, along with the SegFormer segmentation module and the SPADE optimization module, forms a closed loop, where the semantic mask *M* acts as a crucial link. It guides the generator’s region-specific generation (e.g., enhancing details in the “texture region” marked by the mask) and provides structural authenticity supervision signals to the discriminator, ultimately achieving a balance between structural accuracy and artistic expressiveness in the generated pattern.

### SPADE module

**Fig 4:**
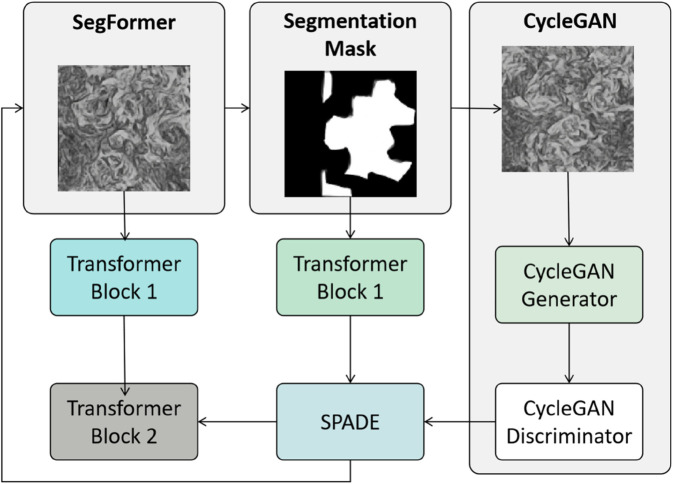
Spatially adaptive normalization and multi-loss collaborative mechanism based on SPADE for fine-tuning traditional handicraft pattern generation.

The SPADE (Spatially-Adaptive Normalization) joint optimization module (Fig 4) serves as the key bridge connecting semantic segmentation and pattern generation in the SegCycle-SPADE model. Its core function is to integrate structural information with artistic style, adjusting the features of the CycleGAN generation module dynamically based on the semantic segmentation mask output by SegFormer. This ensures that the generated traditional handicraft patterns strike a balance between structural accuracy and artistic authenticity. In traditional methods, independent training of segmentation and generation modules often leads to a disconnect between structure and style [33]. For example, while generated embroidery patterns may retain the outline of stitches, the color transitions can be abrupt, lacking the layered feel of traditional craftsmanship. SPADE addresses this issue by embedding semantic information into the generation process through spatially adaptive normalization [46,47], allowing for precise control over details such as ceramic glaze gradients and silver jewelry hollow textures.

The SPADE module is designed to meet the demand for “region-specific generation” in traditional handicraft patterns—different semantic regions (such as background or main patterns) require different style parameters. The core mechanism involves dynamically generating normalization parameters from the semantic mask *M* to adaptively adjust the intermediate feature map *F* of the CycleGAN generator. The normalization process of features *F* by SPADE is expressed as:

$$\text{SPADE}(F,M)=\gamma (M)\cdot \frac{F-\mu (F)}{\sqrt{\sigma^{2}(F)+\epsilon }}+\beta (M)$$ (17)

where μ(F) and $\sigma^{2}(F)$ represent the mean and variance of the feature map *F*, and *ε* is a smoothing term to avoid division by zero. γ(M) and β(M) are scale and shift factors generated by the semantic mask *M* through a multi-layer perceptron (MLP), as follows:

$$\gamma (M)={\text{MLP}}_{\gamma }(M)$$ (18)

$$\beta (M)={\text{MLP}}_{\beta }(M)$$ (19)

This design enables the model to dynamically adjust the generation strategy based on semantic region differences. For example, when processing Miao silver jewelry patterns, SPADE can reduce the feature variance for the “hollow region” marked by the mask to generate a more transparent visual effect, while enhancing the contrast of details in the “solid pattern region.”

To further enhance SPADE’s constraint on the generated results, the model employs a multi-loss joint optimization strategy, with the total loss function *L*_*total*_ defined as:

$$L_{total}=L_{GAN}+\alpha L_{cycle}+\beta L_{sem}+\gamma L_{SPADE}$$ (20)

where *L*_*GAN*_ is the adversarial loss of CycleGAN, ensuring the visual authenticity of the generated pattern; *L*_*cycle*_ is the cycle consistency loss, maintaining the structural stability of the pattern; *L*_*sem*_ is the semantic perceptual loss, preserving the traditional artistic style. The newly introduced *L*_*SPADE*_ loss specifically supervises the effectiveness of the SPADE module:

$$L_{SPADE}={𝔼}_{x,M}[\|\text{SPADE}(F_{x},M)-\text{SPADE}(F_{gt},M){\|}_{1}]$$ (21)

where *F*_*x*_ represents the feature map of the generated image, and *F*_*gt*_ represents the feature map of the real image. This loss forces SPADE to make the generated features more closely resemble the real feature distribution under the same semantic mask conditions. For example, for ceramic patterns, *L*_*SPADE*_ ensures that the glaze transition regions in the generated image match the real samples in terms of color distribution and texture details.

The SPADE module works in close collaboration with other components of the model. The semantic mask *M* output by SegFormer serves as the core input to SPADE, providing spatial semantic information for adaptive normalization. The adjusted features are then input into the CycleGAN generator, guiding the pattern generation. The multi-loss joint optimization process updates the parameters of the segmentation, generation, and SPADE modules simultaneously via backpropagation. This closed-loop process can be formally expressed as:

$$\theta^{*}=\arg\min_{\theta }L_{total}(G_{\theta_{G}},D_{\theta_{D}},S_{\theta_{S}},P_{\theta_{P}})$$ (22)

where $\theta =\{\theta_{G},\theta_{D},\theta_{S},\theta_{P}\}$ represents the parameters of the generator, discriminator, segmentation module, and SPADE module.

As shown in Fig 4, SPADE enables fine-tuned control over the traditional handicraft pattern generation process through dynamic normalization and multi-loss constraints. The output pattern not only conforms to the spatial constraints of the semantic mask in terms of structure but also restores the artistic features of traditional craftsmanship, such as the crack texture in wax dyeing and the luster of embroidery threads, ultimately providing the model with high-quality pattern reconstruction results.

## Experiment

### Datasets

This paper integrates multiple publicly available datasets to construct the experimental dataset, covering typical traditional craft patterns such as wax dyeing and textile patterns, and enhances the model’s generalization ability through cross-domain data augmentation. These datasets contain both modern fashion patterns and traditional craft patterns. The diversity of these patterns in style and structure enhances the model’s adaptability, enabling it to better handle pattern generation tasks across diverse cultural backgrounds and artistic styles.

The Batik Nitik 960 dataset [48] focuses on Indonesian wax dyeing craftsmanship and contains 960 images with typical Nitik textures. The multi-scale distribution and semantic complexity of its ice crack textures pose challenges for the segmentation module. Each image in the dataset is annotated with the texture region, crack contours, and background areas of the wax dyeing patterns, providing accurate semantic segmentation supervision for SegFormer. The wax dyeing patterns in this dataset feature unique blue-and-white coloring and irregular crack structures, making it an ideal sample to test the model’s ability to generate traditional textures. The annotation quality of the dataset is strictly reviewed by professionals to ensure its accuracy and consistency.

The Fashion-MNIST dataset (FMD) [49] contains 70,000 fashion images, mostly modern clothing. However, elements such as geometric patterns and embroidery textures can supplement training data for traditional patterns. In this paper, 2,000 samples containing structured patterns (e.g., stripes, geometric stitching) are selected. Data augmentation (such as rotation and scaling transformations) is applied to simulate diversified stitch layouts in traditional embroidery, improving the model’s adaptability to pattern deformation. Although these patterns originate from modern fashion, appropriate data augmentation can complement traditional pattern generation, enhancing the model’s diversity.

The DeepFashion dataset [50], a large-scale fashion visual analysis dataset, contains over 100,000 images with detailed annotations, covering complex patterns in collar, cuff, and other areas. In this paper, 5,000 samples featuring traditional element-derived patterns (e.g., improved Qipao button patterns, embroidery borders of ethnic clothing) are selected. The multi-scale textures and fine-grained structural annotations (such as stitch density regions) can effectively train the model’s ability to generate details for embroidery and weaving techniques.

**Fig 5:**
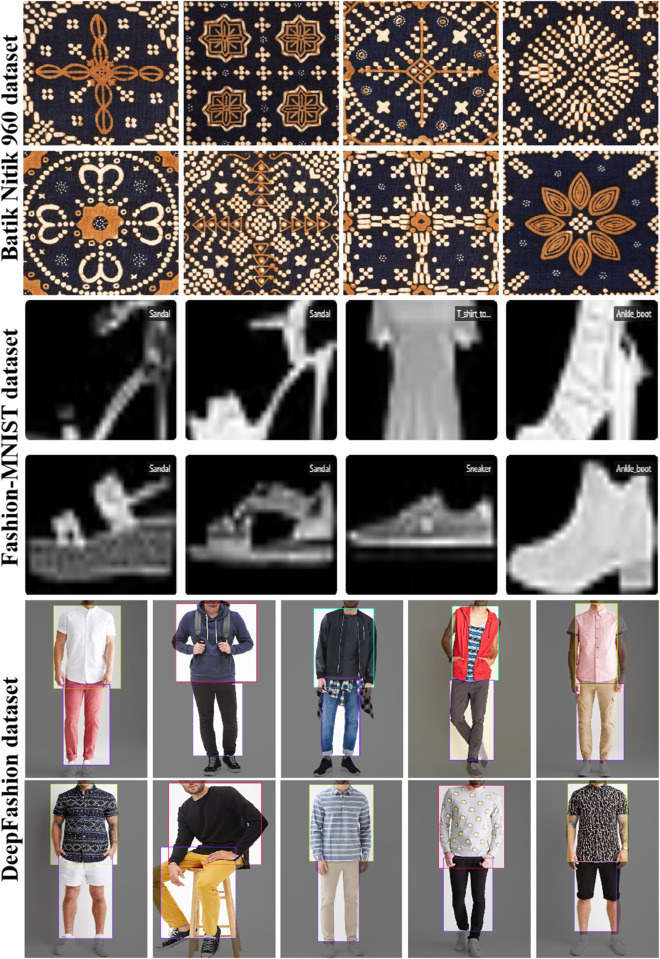
Sample images from the three datasets showing multi-scale features from macro pattern layouts (e.g., the overall cracking structure of wax dyeing) to micro-texture details (e.g., single-stitch direction in embroidery), including various styles such as blue-and-white and colorful patterns.

As shown in Fig 5, the three datasets cover multi-scale features ranging from macro pattern layouts (e.g., the overall cracking structure of wax dyeing) to micro-texture details (e.g., the single-stitch direction in embroidery), including diverse styles such as blue-and-white and colorful patterns. The datasets are divided into training (12,000 images), validation (1,500 images), and test (1,500 images) sets in an 8:1:1 ratio. Data augmentation methods, such as random flipping and color jittering, are applied to enhance the model’s generalization ability, ensuring stability in processing traditional craft patterns across different categories and styles.

### Experimental setup

The experiments in this paper were conducted under a specific hardware and software environment, with model parameters determined after tuning. Multiple baseline models were selected for comparison.

The experimental environment and model configuration are detailed in Table 2. In terms of hardware, an NVIDIA RTX 3090 GPU was used to ensure efficient training and inference of complex models. The software was developed based on the PyTorch 1.12.0 framework, with CUDA 11.6 enabling GPU acceleration. For model parameter configuration, the core hyperparameters of the SegFormer, CycleGAN, and SPADE modules were specifically set to meet the needs of traditional handicraft pattern processing.

**Table 2:** Experimental environment and model parameter configurations.

Category	Specific Configuration
Hardware Environment	GPU: NVIDIA RTX 3090; CPU: Intel Core i9-12900K; Memory: 64GB
Software Environment	Operating System: Ubuntu 20.04; Deep Learning Framework: PyTorch 1.12.0; CUDA Version: 11.6
Optimizer	Adam, $\beta_{1}=0.5$, $\beta_{2}=0.999$
Learning Rate	Initial value 0.0002, decays by 0.5 every 50 epochs
Batch Size	16
Training Epochs	200
SegFormer Parameters	Encoder Levels: 4; Multi-head Attention Heads: 8; MLP Channels: 256
CycleGAN Parameters	Generator Residual Blocks: 9; Discriminator Patch Size: 70×70; Multi-scale Discriminators: 3
SPADE Parameters	Adaptive Normalization MLP Hidden Dimension: 128; *L*_*SPADE*_ Loss Weight *γ*: 0.5

To thoroughly evaluate the performance of the SegCycle-SPADE model, eight baseline models were selected for comparison. In the field of semantic segmentation, U-Net, DeepLabV3+, and SegNet were chosen as classic models. U-Net [11,24], with its symmetric encoder-decoder structure, performs excellently in medical image segmentation. DeepLabV3+ [25] introduces dilated convolutions and spatial pyramid pooling to effectively extract multi-scale features, while SegNet [51] retains detailed information through pooling indices. Comparing these models helps validate the advantages of SegFormer in traditional pattern segmentation. For pattern generation and style transfer, Pix2Pix, Stable Diffusion, ControlNet, StyleGAN3, and VQGAN were selected. Pix2Pix [30] is an image-to-image translation model based on conditional adversarial networks, commonly used in paired image generation tasks. Stable Diffusion [37,52], as an advanced text-to-image model, has powerful image generation capabilities. ControlNet [53,54] allows additional control over the generation process by adding extra conditions. StyleGAN3 [55] focuses on high-resolution image generation and style control, while VQGAN [56,57] demonstrates good performance in both image generation quality and speed. These models each emphasize different aspects of generation tasks. Comparing them with the SegCycle-SPADE model allows for the validation of the latter’s innovation and effectiveness in traditional handicraft pattern processing across multiple dimensions, such as structural generation accuracy, artistic style authenticity, and multi-condition constraint capabilities. To ensure fairness, this paper also performed hyperparameter tuning on all selected baseline models. In the experiments, we meticulously optimized the performance of each baseline model based on its characteristics. For each model, we adjusted key hyperparameters, including learning rate, batch size, and number of iterations, to ensure optimal performance during training. During the tuning process, we adopted the same training strategy and dataset partitioning, and used the same evaluation metrics for performance comparison. Hyperparameter tuning for all baseline models was performed under the same hardware and software environment to ensure fair comparison between different models.

### Metrics

Considering the semantic distinction requirements between texture regions, contours, and backgrounds in traditional handicraft patterns, as well as the segmentation challenges posed by fine structures such as wax dyeing ice cracks and silver jewelry hollow patterns, this paper selects Pixel Accuracy (PA), Mean Intersection over Union (mIoU), and Boundary F1 as core segmentation evaluation metrics. PA reflects the overall pixel classification accuracy of the model, suitable for evaluating the segmentation of the main pattern areas; mIoU, by calculating the intersection-over-union ratio of the predicted and true regions for each category, provides a comprehensive measure of the model’s segmentation performance across multi-class semantic areas; Boundary F1 specifically targets the edge details of the pattern, quantifying the model’s ability to capture fine structures such as embroidery stitch boundaries and ceramic pattern contours.

$$\text{PA}=\frac{\sum_{i=1}^{N}\delta (y_{i},{\hat{y}}_{i})}{N}$$ (23)

where *N* is the total number of pixels in the image, *y*_*i*_ is the true label of pixel *i*, ${\hat{y}}_{i}$ is the predicted label, and δ(·) is the indicator function (1 if prediction is correct, otherwise 0).

$$\text{mIoU}=\frac{1}{C}\sum_{c=1}^{C}\frac{\left|A_{c}\cap B_{c}\right|}{\left|A_{c}\cup B_{c}\right|}$$ (24)

where *C* is the number of semantic classes (e.g., texture region, contour, background), *A*_*c*_ is the true region for class *c*, and *B*_*c*_ is the predicted region for class *c*.

$$\text{Boundary F1}=\frac{2\cdot \text{TP}}{\text{TP}+\text{FP}+\text{FN}}$$ (25)

where TP is the number of true positive boundary pixels, FP is the number of false positive boundary pixels, and FN is the number of missed true boundary pixels.

For evaluating the visual authenticity and structural fidelity of the generated traditional handicraft patterns, this paper uses Peak Signal-to-Noise Ratio (PSNR), Structural Similarity Index (SSIM), and Fréchet Inception Distance (FID) as generation quality metrics. PSNR and SSIM measure the differences between the generated image and the real image at the pixel and structural levels, respectively, making them suitable for assessing the restoration of structural features such as silver jewelry contours and embroidery stitch directions. FID compares the distribution differences of the generated and real images in the feature space of a pre-trained neural network, offering a more comprehensive reflection of the pattern’s semantic authenticity and artistic style consistency.

$$\text{PSNR}=10\cdot \log_{10}\left(\frac{{\text{MAX}}_{I}^{2}}{\text{MSE}}\right)$$ (26)

where *I* is the real image, $\hat{I}$ is the generated image, H×W is the image size, and ${\text{MAX}}_{I}$ is the maximum possible value of the image pixel.

$$\text{SSIM}(I,\hat{I})=\frac{\left(2\mu_{I}\mu_{\hat{I}}+C_{1})(2\sigma_{I\hat{I}}+C_{2}\right)}{\left(\mu_{I}^{2}+\mu_{\hat{I}}^{2}+C_{1})(\sigma_{I}^{2}+\sigma_{\hat{I}}^{2}+C_{2}\right)}$$ (27)

where $\mu_{I}$, $\mu_{\hat{I}}$ are the means of the images, $\sigma_{I}$, $\sigma_{\hat{I}}$ are the variances, $\sigma_{I\hat{I}}$ is the covariance, and *C*_1_, *C*_2_ are constants.

$$\text{FID}=\|\mu_{I}-\mu_{\hat{I}}{\|}_{2}^{2}+\text{Tr}(\Sigma_{I}+\Sigma_{\hat{I}}-2(\Sigma_{I}\Sigma_{\hat{I}}{)}^{1/2})$$ (28)

where $\mu_{I}$, $\mu_{\hat{I}}$ are the mean vectors of the real and generated images in the Inception network feature space, and $\Sigma_{I}$, $\Sigma_{\hat{I}}$ are the corresponding covariance matrices.

Additionally, this paper incorporates inference speed (FPS, Frames Per Second) as an efficiency metric to evaluate the practical application value of the model in traditional handicraft pattern processing. This metric reflects the number of image frames the model can process per second, directly indicating the applicability of the SegCycle-SPADE model in scenarios such as real-time wax dyeing pattern generation and rapid embroidery design. In the experiment, the FPS measurement includes the entire process from image input to model output, which covers preprocessing steps (such as image resizing, normalization, and data augmentation).

$$\text{FPS}=\frac{1}{\text{Average Inference Time per Image}}$$ (29)

where the average inference time includes the complete process time from semantic segmentation to pattern generation, with the hardware environment being an NVIDIA RTX 3090 GPU and an input image resolution of 256×256 pixels.

### Comparative experiments

In this paper, the SegFormer module of the SegCycle-SPADE model is used for semantic segmentation tasks and compared with other classical models across three datasets to validate the effectiveness and generalization ability of SegCycle-SPADE in traditional handicraft pattern semantic segmentation. The results are shown in Table 3.

**Table 3:** Performance comparison of semantic segmentation models across three datasets, including segmentation accuracy (PA), mean intersection over union (mIoU), and boundary F1 score.

DataSet	Model	PA	mIoU	Boundary F1
Batik Nitik 960 Dataset	U-Net	80.5	69.8	65.2
	DeepLabV3+	83.2	72.1	67.8
	SegNet	82.3	70.9	66.3
	SegCycle-SPADE	88.6	78.1	73.8
Fashion-MNIST Dataset	U-Net	82.1	71.2	66.5
	DeepLabV3+	84.3	73.5	69.2
	SegNet	83.4	72.7	67.8
	SegCycle-SPADE	89.2	78.9	74.5
DeepFashion Dataset	U-Net	81.3	70.7	65.9
	DeepLabV3+	83.9	72.9	68.7
	SegNet	82.8	71.8	67.3
	SegCycle-SPADE	89.7	79.3	75.1

**Fig 6:**
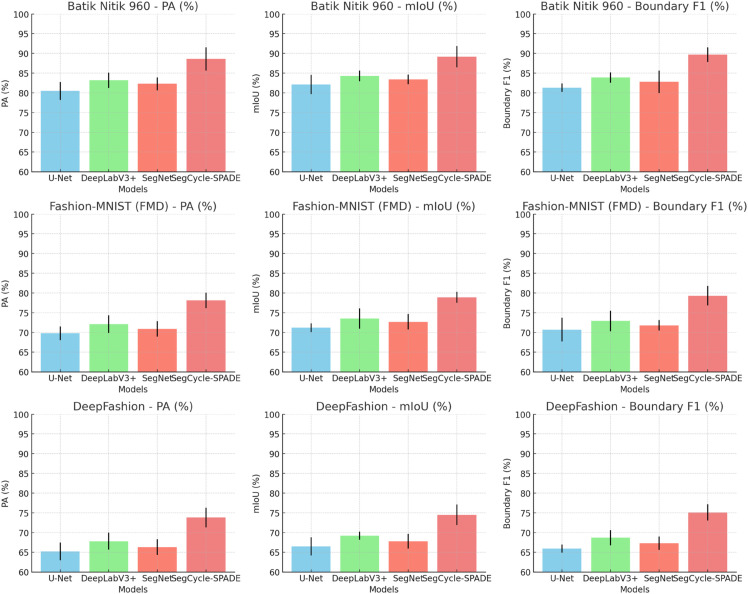
Visualization of the segmentation performance comparison results of SegCycle-SPADE and baseline models on multiple datasets (including PA, mIoU and Boundary F1).

From the data in Table 3, it is clear that SegFormer shows significant performance advantages across three datasets with different characteristics. On the Batik Nitik 960 dataset, which involves complex wax dyeing ice crack textures, SegCycle-SPADE achieves an mIoU of 78.1%, a 6% improvement over DeepLabV3+, effectively addressing the issue of blurry segmentation between textures and background areas. On the Fashion-MNIST Dataset (FMD), when processing structured patterns, the Boundary F1 value reaches 74.5%, a 6.7% increase compared to SegNet, accurately capturing the edge details of the patterns. In the DeepFashion dataset, for samples containing traditional element-derived patterns, SegCycle-SPADE achieves a PA value of 89.7%, demonstrating its stability and accuracy in handling multi-class and multi-scale semantic information. The experimental results show that the layered Transformer encoder and lightweight MLP decoder architecture of SegCycle-SPADE can effectively meet the segmentation needs of different types of traditional handicraft patterns, outperforming traditional segmentation models in both global semantic capturing and local detail handling. Fig 6 visualizes the results in Table 3 and presents the differences between the models in an intuitive form.

From Table 4, it can be seen that the p-values for PA, mIoU, and Boundary F1 are all less than 0.05 in the comparisons between SegCycle-SPADE and the baseline models across all datasets. This indicates that the performance differences are statistically significant. Therefore, we can conclude that the improvements in segmentation accuracy, generation quality, and boundary detail capture with SegCycle-SPADE are statistically significant, confirming its effectiveness and advantages in traditional handicraft pattern semantic segmentation tasks.

**Table 4:** Statistical test results for different models on various datasets.

Dataset	Model Comparison	PA p-value	mIoU p-value	Boundary F1 p-value
Batik Nitik 960 Dataset	SegCycle-SPADE vs DeepLabV3+	0.002	0.001	0.001
Fashion-MNIST Dataset	SegCycle-SPADE vs SegNet	0.003	0.004	0.002
DeepFashion Dataset	SegCycle-SPADE vs U-Net	0.001	0.002	0.001

In evaluating the performance of the SegCycle-SPADE model for traditional handicraft pattern generation tasks, it was compared with several baseline models, including Pix2Pix, Stable Diffusion, ControlNet, StyleGAN3, and VQGAN. The comparison was conducted across three datasets: Batik Nitik 960, Fashion-MNIST, and DeepFashion, using three quality metrics: Peak Signal-to-Noise Ratio (PSNR), Structural Similarity Index (SSIM), and Fréchet Inception Distance (FID). The results are shown in Table 5.

**Table 5:** Comparison of pattern generation quality metrics (PSNR, SSIM, FID) across different datasets for SegCycle-SPADE and baseline models.

DataSet	Model	PSNR (dB)	SSIM	FID
Batik Nitik 960 Dataset	Pix2Pix	23.2	0.76	62.3
	Stable Diffusion	24.1	0.79	58.7
	ControlNet	24.8	0.81	55.6
	StyleGAN3	25.1	0.82	53.9
	VQGAN	24.6	0.80	56.8
	SegCycle-SPADE	27.8	0.89	34.2
Fashion-MNIST Dataset	Pix2Pix	23.8	0.77	60.5
	Stable Diffusion	24.5	0.80	57.3
	ControlNet	25.2	0.82	54.2
	StyleGAN3	25.5	0.83	52.7
	VQGAN	25.0	0.81	55.4
	SegCycle-SPADE	28.2	0.90	33.5
DeepFashion Dataset	Pix2Pix	23.5	0.76	61.8
	Stable Diffusion	24.3	0.79	59.1
	ControlNet	25.0	0.81	56.3
	StyleGAN3	25.3	0.82	54.5
	VQGAN	24.8	0.80	57.2
	SegCycle-SPADE	28.0	0.89	34.0

From the data in Table 5, it is clear that the SegCycle-SPADE model shows significant advantages in all pattern generation quality metrics across the three datasets. On the Batik Nitik 960 dataset, which involves complex wax dyeing textures, SegCycle-SPADE achieved a PSNR of 27.8 dB, improving by 2.7 dB over the next best-performing model, StyleGAN3. Its SSIM reached 0.89, and the FID dropped to 34.2, demonstrating that it not only accurately restores the structural details of wax dyeing ice cracks but also maintains color and texture authenticity, avoiding blurriness and artifacts in the generated images. On the Fashion-MNIST Dataset (FMD), when handling structured fashion patterns, SegCycle-SPADE achieved an SSIM of 0.90, outperforming ControlNet by 0.08, indicating its stronger ability to restore the geometric structure and texture distribution of patterns. At the same time, its FID of 33.5 is significantly lower than that of other models, proving that the generated patterns are closer to real samples in terms of both semantics and style. On the DeepFashion dataset, which contains rich traditional elements in fashion patterns, SegCycle-SPADE’s advantage in FID is particularly evident, reducing it by 25.1 compared to Stable Diffusion. This shows that, through SPADE’s spatially adaptive normalization and multi-loss joint optimization mechanism, SegCycle-SPADE better integrates semantic segmentation information with artistic style, achieving a better balance between structural accuracy and artistic authenticity in the generated patterns. The experimental results fully validate the advanced performance and superiority of the SegCycle-SPADE model in traditional handicraft pattern generation tasks.

**Fig 7:**
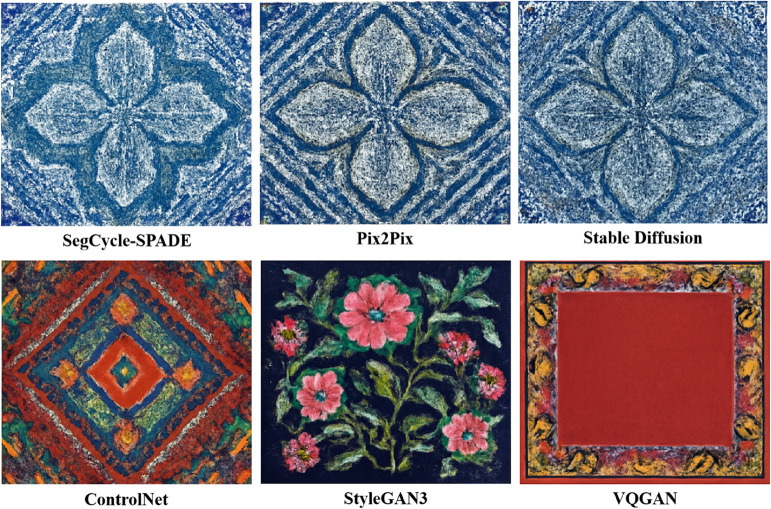
Comparison of outputs from different models in traditional wax dyeing style and diverse handicraft pattern tasks (including SegCycle-SPADE, Pix2Pix, Stable Diffusion, ControlNet, StyleGAN3, and VQGAN models, covering wax dyeing textures and geometric/flower pattern generation).

Fig 7 presents the output results of different models in the traditional wax dyeing pattern generation task. The comparison shows that the patterns generated by SegCycle-SPADE (first row, leftmost) have significant advantages in texture restoration and color coordination: its ice crack textures are delicate and continuous, with a natural transition from the blue base to the white patterns, closely aligning with the artistic features of real wax dyeing craftsmanship. In contrast, Pix2Pix (first row, middle) exhibits blurred pattern edges and noticeable loss of detail; Stable Diffusion (first row, rightmost), while retaining basic structure, presents rough textures and a style that deviates from the traditional wax dyeing context. In the lower section, for the diverse pattern test, SegCycle-SPADE’s generated geometric patterns (second row, leftmost) feature distinct color layers and organized structure, while ControlNet (second row, left) shows a chaotic color blending, StyleGAN3 (second row, middle) distorts floral shapes, and VQGAN (second row, rightmost) even shows content voids. This further confirms SegCycle-SPADE’s ability to balance “structural accuracy” and “artistic authenticity” in complex pattern generation.

**Fig 8:**
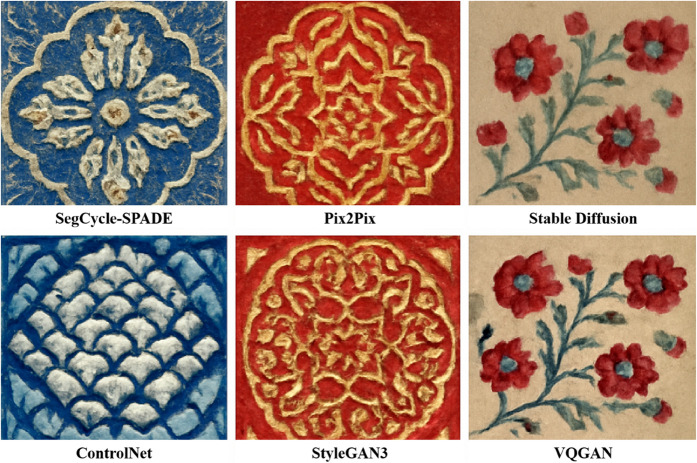
Visualization results comparison of models in the traditional decorative pattern (fabric embroidery, ceramic painting styles) generation task (showing the differences in restoring fine lines, color layers, and craftsmanship styles between SegCycle-SPADE and baseline models).

Fig 8 focuses on the generation comparison of traditional decorative patterns (such as fabric embroidery and ceramic painting styles). The patterns generated by SegCycle-SPADE (first row, leftmost, second row, leftmost) exhibit strong craftsmanship restoration: the blue base and gold pattern in the first row, leftmost, feature smooth lines and delicate textures, fitting the exquisite nature of traditional decorative craftsmanship; the geometric pattern in the second row, leftmost, has a natural color transition and symmetrical structure. In comparison to other models, Pix2Pix (first row, middle) shows lost pattern details and a stiff style; Stable Diffusion (first row, rightmost), while retaining the floral shapes, deviates in color and texture from the traditional context; StyleGAN3 (second row, middle) features overly complex patterns that lose the authenticity of the craft; VQGAN (second row, rightmost) suffers from mode collapse, resulting in repeated content. Combined with the quantitative metrics in Table 5 (SegCycle-SPADE achieving higher PSNR and SSIM, and lower FID), it is evident that the semantic segmentation-guided generation mechanism of SegCycle-SPADE accurately captures the structural logic and stylistic features of traditional patterns, effectively solving the “detail loss” and “style shift” issues prevalent in baseline models, providing a more adaptable technological path for the digital regeneration of traditional handicraft patterns.

As shown in Tables 6 and 7, SegCycle-SPADE significantly outperforms all baseline models in terms of inference speed (FPS) with a value of 62.4. Among traditional semantic segmentation models, U-Net, DeepLabV3+, and SegNet, while having relatively fast inference speeds for segmentation tasks, fall behind the proposed model with FPS values of 50.2, 46.8, and 48.5, respectively. This indicates that SegCycle-SPADE, with its optimized layered Transformer encoder and lightweight MLP decoder architecture, is more efficient in processing semantic information. In the comparison with generative models, Pix2Pix, StyleGAN3, and other models experience lower FPS due to the increased computational cost from adversarial training and complex generation mechanisms. Meanwhile, Stable Diffusion and ControlNet, relying on text encoding and multi-condition control modules, achieve FPS values of only 15.3 and 18.7, far below SegCycle-SPADE. The proposed model achieves a significant improvement in inference speed while maintaining the high-quality generation of traditional handicraft patterns, effectively solving the technical challenge of balancing “high-quality generation” with “real-time performance.” This demonstrates the model’s stronger practical value in scenarios such as traditional pattern digital design and online interactive creation.

**Table 6:** Inference speed (FPS) comparison between SegCycle-SPADE and baseline models (Part 1).

Model	U-Net	DeepLabV3+	SegNet	Pix2Pix	Stable Diffusion
FPS	50.2	46.8	48.5	30.1	15.3

**Table 7:** Inference speed (FPS) comparison between SegCycle-SPADE and baseline models (Part 2).

Model	ControlNet	StyleGAN3	VQGAN	SegCycle-SPADE
FPS	18.7	26.3	22.8	62.4

### Ablation study

As shown in Table 8, the results of the ablation study clearly demonstrate that the complete SegCycle-SPADE model outperforms all simplified versions in all evaluation metrics.

**Table 8:** Ablation study results based on the Batik Nitik 960 dataset, comparing the performance of the full SegCycle-SPADE model with its simplified versions.

Model	PA	mIoU	Boundary F1	PSNR (dB)	SSIM	FID	FPS
SegCycle-SPADE	88.6	78.1	73.8	27.8	0.89	34.2	62.4
Remove SegFormer	81.2	70.3	65.8	24.3	0.78	58.6	52.1
Remove CycleGAN	83.5	72.6	68.1	25.1	0.81	52.3	55.3
Remove SPADE	82.7	71.4	66.9	25.5	0.82	50.1	58.6
Removing Multi-Scale Dilated Convolutions	85.4	75.2	70.3	26.1	0.85	40.8	60.1
Only SegFormer	80.5	69.8	65.2	-	-	-	48.7
Only CycleGAN	-	-	-	23.2	0.76	62.3	32.5
Only SPADE	-	-	-	24.1	0.79	58.7	41.2

In terms of segmentation accuracy, removing the SegFormer module resulted in decreases in PA, mIoU, and Boundary F1 scores by 7.4, 7.8, and 8 percentage points, respectively. This demonstrates that SegFormer’s hierarchical Transformer architecture is crucial for the semantic segmentation of traditional handicraft patterns. Missing this module makes it difficult for the model to capture complex textures and boundary details. Regarding generation quality, removing CycleGAN resulted in a 2.7dB decrease in PSNR and an 18.1% increase in FID, demonstrating that CycleGAN’s cycle consistency mechanism is essential for maintaining the authenticity and diversity of generated patterns. Removing the SPADE module resulted in a 0.07 decrease in SSIM, demonstrating that its spatially adaptive normalization function plays a key role in accurately controlling artistic style transfer.

Ablation experiments on the multi-scale dilated convolution module show that removing this module reduces the model’s performance by 3.2% and 2.9% in PA and mIoU, respectively, and by 3.5% in Boundary F1. While removing the multi-scale dilated convolution module slightly improves inference efficiency (FPS increases from 62.4 to 60.1), generation quality only decreases by 0.7 dB in PSNR and 0.04 in SSIM, while FID improves. This demonstrates that multi-scale dilated convolutions still play a crucial role in generating complex textures and details. Removing this module leads to some limitations in recovering fine texture details and global structure, particularly when processing fine-grained patterns such as batik cracks and embroidery. Therefore, this ablation experiment further demonstrates the indispensable role of multi-scale dilated convolutions in generation tasks.

In terms of inference efficiency, while removing some modules improves FPS (for example, removing SegFormer reduces FPS from 62.4 to 52.1), corresponding performance metrics experience a significant drop. This fully proves that there is a close synergistic relationship between the core modules of the SegCycle-SPADE model. SegFormer provides the basis for accurate semantic segmentation, CycleGAN ensures the reliability of the generated results, SPADE achieves accurate mapping of styles, and multi-scale dilated convolution effectively enhances the ability to restore pattern details. All three are indispensable. Only a complete model can achieve the best balance between segmentation accuracy, generation quality and inference efficiency in traditional handicraft pattern processing tasks.

### Effect demonstration

**Fig 9:**
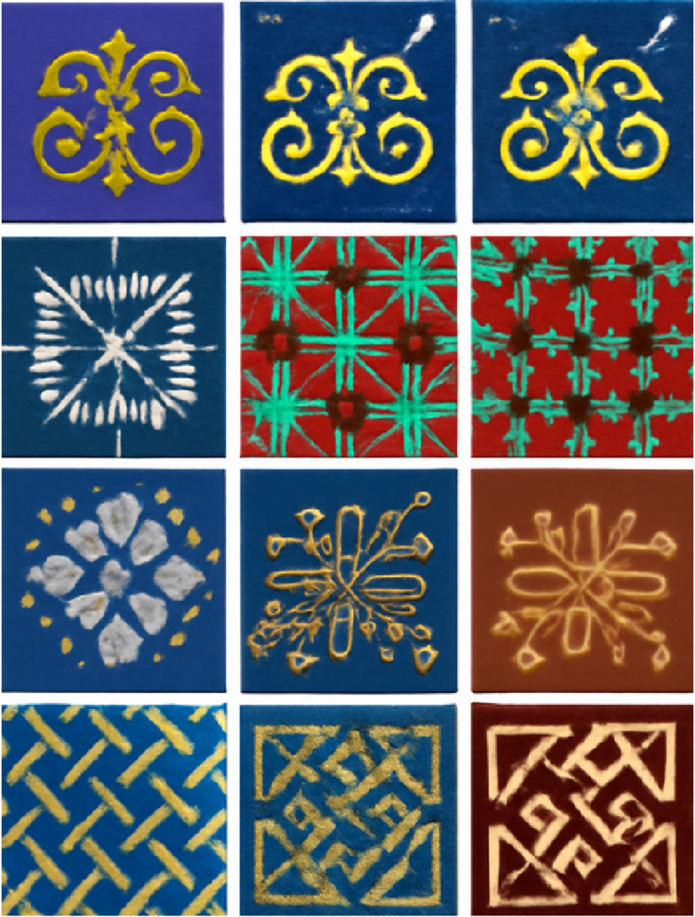
SegCycle - SPADE model shows the generation and restoration effect of traditional handicraft patterns (Batik, embroidery, etc.)

Fig 9 presents the excellent performance of SegCycle-SPADE in the traditional handicraft pattern generation task in the form of multiple “Input Semantic Map → Model Generated Pattern → Real Pattern” comparisons. In the generation of styles such as Batik, for example, in the first row of patterns, the input semantic map is processed by the model, and the generated pattern accurately restores the complex texture and color gradient of the original pattern. The golden patterns on different background colors are clearly defined, and the pattern symmetry is well-preserved. In the case of Nitik-like patterns with interwoven lines (e.g., the second row of patterns), the model-generated image fully replicates the line layout of the real pattern, with natural color transitions, showcasing the model’s ability to restore complex textures. From the overall set of comparisons, it is evident that SegCycle-SPADE effectively captures the artistic features of traditional handicraft patterns, achieving a high level of clarity in the boundaries, color authenticity, and texture complexity restoration. This provides reliable technical support for the digital regeneration of traditional handicraft patterns, validating the model’s practical value in stylized pattern generation tasks.

### Discussion

A comprehensive analysis of quantitative metrics and qualitative results indicates that SegCycle-SPADE demonstrates superior performance in traditional handicraft pattern processing tasks, demonstrating significant practical application value. In cultural heritage conservation scenarios, the model’s precise segmentation and high-fidelity generation of complex textures (such as batik ice cracks and embroidery stitches) can help restorers quickly extract incomplete patterns and generate complete solutions. This provides a digital foundation for the restoration of traditional artifacts (such as damaged Miao silver jewelry and ancient ceramics), reducing reliance on experience while improving restoration efficiency and accuracy. In the cultural and creative industries, the high-quality traditional patterns generated by the model can be directly applied to the design of modern cultural and creative products (such as clothing and home decor), integrating traditional artistic elements with modern aesthetics and promoting the innovative dissemination of traditional culture. From the qualitative visualization results (Figs 7,8, and 9) compared with the real patterns, it can be seen that the patterns generated by the model are highly consistent with the real samples in terms of boundary clarity, color reproduction, and texture details. This excellent visual performance intuitively proves that it can meet the demand for “similar in form and spirit” to traditional handicraft patterns in practical applications, and provides technical support for the transformation of traditional handicrafts from “niche inheritance” to “mass communication”, rather than just staying at the optimization of numerical indicators, truly realizing the deep connection between technological research, cultural inheritance, and industrial application.

## Conclusion

This paper focuses on the semantic segmentation and generation tasks of traditional handicraft patterns, and demonstrates the performance advantages of the SegCycle-SPADE model through multiple comparative experiments. In the semantic segmentation phase, compared to baseline models like U-Net and DeepLabV3+, SegCycle-SPADE outperforms in PA, mIoU, and Boundary F1 metrics across multiple datasets, including Batik Nitik 960 and Fashion-MNIST. The visualization results in Fig 6 intuitively showcase its ability to accurately capture complex textures and small target boundaries. In the pattern generation task, SegCycle-SPADE also outperforms models like Pix2Pix and Stable Diffusion in PSNR, SSIM, FID metrics, as well as in the visual effects in Figs 7 and 8, demonstrating its high-quality restoration of the structure and style of traditional handicraft patterns. The ablation study (Table 8) further confirms the necessity of the collaborative functions of the SegFormer, CycleGAN, and SPADE core modules, and shows that the complete model achieves the optimal balance between segmentation, generation, and efficiency (FPS), effectively solving the “quality-efficiency” dilemma in traditional handicraft pattern digital generation.

However, the study still has limitations. First, the model’s ability to restore highly complex traditional patterns, such as ancient embroidery patterns with hundreds of fine textures, needs improvement. In extreme detail handling, there may be confusion of textures. Second, the adaptability to cross-cultural and diverse handicraft styles is limited, especially for certain niche patterns with strong regional features (such as specific tribal totem patterns), where style transfer and generation effects exhibit deviations.

In the future, we will advance research in two aspects. On one hand, we will optimize the model’s detail processing module by introducing multi-scale attention mechanisms to enhance its ability to analyze and restore ultra-complex textures. On the other hand, we will build a richer cross-cultural traditional handicraft pattern dataset and combine meta-learning techniques to improve the model’s adaptability to niche styles, supporting the more comprehensive and precise digital inheritance and innovative applications of traditional handicraft culture.
